# Aberrant *N*-Glycosylation Profile of Serum Immunoglobulins is a Diagnostic Biomarker of Urothelial Carcinomas

**DOI:** 10.3390/ijms18122632

**Published:** 2017-12-06

**Authors:** Toshikazu Tanaka, Tohru Yoneyama, Daisuke Noro, Kengo Imanishi, Yuta Kojima, Shingo Hatakeyama, Yuki Tobisawa, Kazuyuki Mori, Hayato Yamamoto, Atsushi Imai, Takahiro Yoneyama, Yasuhiro Hashimoto, Takuya Koie, Masakazu Tanaka, Shin-Ichiro Nishimura, Shizuka Kurauchi, Ippei Takahashi, Chikara Ohyama

**Affiliations:** 1 Department of Urology, Hirosaki University Graduate School of Medicine, Hirosaki 036-8562, Japan; yosage1205@yahoo.co.jp (T.T.); noro.daisuke@camel.plala.or.jp (D.N.); born_2b_snower@yahoo.co.jp (K.I.); y_kojima0319@yahoo.co.jp (Y.K.); shingoh@hirosaki-u.ac.jp (S.H.); tobisawa@hirosaki-u.ac.jp (Y.T.); moribio@hirosaki-u.ac.jp (K.M.); yamahaya10@yahoo.co.jp (H.Y.); tsushi.imai@gmail.com (A.I.); uroyone@hirosaki-u.ac.jp (T.Y.); goodwin@hirosaki-u.ac.jp (T.K.); coyama@hirosaki-u.ac.jp (C.O.); 2 Department of Advanced Transplant and Regenerative Medicine, Hirosaki University Graduate School of Medicine, Hirosaki 036-8562, Japan; bikkuri@opal.plala.or.jp; 3 Graduate School of Life Science, Frontier Research Centre for Advanced Material and Life Science, Hokkaido University, Sapporo 060-0810, Japan; tanaka@soyaku.co.jp (M.T.); shin@sci.hokudai.ac.jp (S.-I.N.); 4 Department of Social Medicine, Hirosaki University Graduate School of Medicine, Hirosaki 036-8562, Japan; k-shizu@hirosaki-u.ac.jp (S.K.); ippei@hirosaki-u.ac.jp (I.T.)

**Keywords:** diagnostic biomarker, urothelial carcinoma of the bladder, upper urinary tract urothelial carcinoma, *N*-glycomics, immunogloburins, aberrant *N*-glycosylation

## Abstract

The aim of this study to determine whether the aberrant *N*-glycosylated serum immunoglobulins (Igs) can be applied as a diagnostic marker of urothelial carcinoma (UC). Between 2009 and 2016, we randomly obtained serum available from 237 UC and also 96 prostate cancer as other cancer controls from our serum bank and also obtained—from 339 healthy volunteers (HV)—controls obtained from community-dwelling volunteers in Iwaki Health Promotion Project. A total of 32 types of *N*-glycan levels on Igs were determined by high-throughput *N*-glycomics and analyzed by multivariable discriminant analysis. We found five UC-associated aberrant *N*-glycans changes on Igs and also found that asialo-bisecting GlcNAc type *N*-glycan on Igs were significantly accumulated in UC patients. The diagnostic *N*-glycan Score (d*N*GScore) established by combination of five *N*-glycans on Igs discriminated UC patients from HV and prostate cancer (PC) patients with 92.8% sensitivity and 97.2% specificity. The area under the curve (AUC) for of the d*N*GScore was 0.969 for UC detection that was much superior to that of urine cytology (AUC, 0.707) and hematuria (AUC, 0.892). Furthermore, d*N*GScore can detect hematuria and urine cytology negative patients. The d*N*Gscore based on aberrant *N*-glycosylation signatures of Igs were found to be promising diagnostic biomarkers of UCs.

## 1. Introduction

Urothelial carcinomas (UCs) are the eighth-most lethal cancer in men in the United States [1]. The majority of UCs originate from bladder, called UC of the bladder (UCB), and between 5% and 10% of UCs originate from the ureter or renal pelvis [2,3], which are collectively called upper urinary tract UCs (UTUCs), with a worse prognosis than that of UCB. The most common symptom is visible- or non-visible hematuria (70–80%) [4,5] and then the standard examinations are performed, involving urine cytology, urinary tract imaging and cystoscopy, which are powerful diagnostic tools for UCs. However, 60% of UTUCs are invasive at time of diagnosis [6,7]. Urine cytology is not reliable in patients with early stage UCs, including UTUC, and it is difficult to visualise small tumors via imaging modalities, such as ultrasound or computed tomography. Several diagnostic urine-based biomarkers are reported such as bladder tumor antigen (BTA), nuclear matrix protein number 22 (NMP22) and UroVysion [8,9,10]. Although sensitivities can be better for BTA (50–80%), NMP22 (68.5–88.5%) and UroVysion (58–96%) over urine cytology (50–67%), the specificities are quite low. Because those tests are soluble antigen test or cellular assay depending on the amount of tumor cells, they are not suitable for the detection of low-grade and low-stage tumors. Furthermore, the prevalence of urinary markers of only about 30% is not enough to complement urine cytology and other invasive tests. Thus, more sensitive and non-invasive biomarkers, such as serum-based biomarkers, to avoid under-detection in patients at high risk of UCs is required.

Glycosylation is a common post-translational modification that has an important role in various biological functions. Previously, our group demonstrated that high-throughput, comprehensive and quantitative *N*-glycomics based on the glycoblotting method combined with mass spectrometry is a promising way to screen glycans for use as diagnostic and prognostic markers of several cancer [11,12,13,14,15]. Recently, the evaluation of glycosylation profile is an innovative topic in different cancer types, especially in immunotherapeutic targets, as like as PD-L1 [16,17,18]. Most recently, our group reported that a combination of several serum *N*-glycans (*N*-glycan score, *N*GScore) is a novel serum marker for UCs including UTUC that detected 93% of UC patients and is therefore far more specific than classic urine cytology [19]. We hypothesized that these serum aberrant *N*-glycan change is derived from serum major *N*-glycosylated proteins, such as immunoglobulins (Igs). Although differences in Ig glycosylation are mainly described in immune system-related diseases [20,21,22,23], there are several papers describing aberrant glycosylation of IgG in cancer such as prostate cancer, gastric cancer and colorectal cancer [24,25,26,27]. In addition, aberrant *N*-glycosylation of immunoglobulins in UC has not been reported elsewhere. Thus, in the present study, we performed *N*-glycomics of serum Igs fractions between healthy volunteers (HVs), prostate cancer (PC) and UCs patients to identify the UC-specific aberrant *N*-glycosylated Igs. Furthermore, for clinical applications, we established a diagnostic *N*GScore (d*N*GScore) based on a combination of five *N*-glycans of Igs associated with detection of UCs.

## 2. Results

### 2.1. Downregulation of Asialo Biantennary Type N-Glycans and Accumulation of Asialo-Bisecting GlcNAc with Core Fucosylated N-Glycan on Igs May Occur as a UC-Associated Aberrant N-Glycosylation of Igs

**Figure A1:**
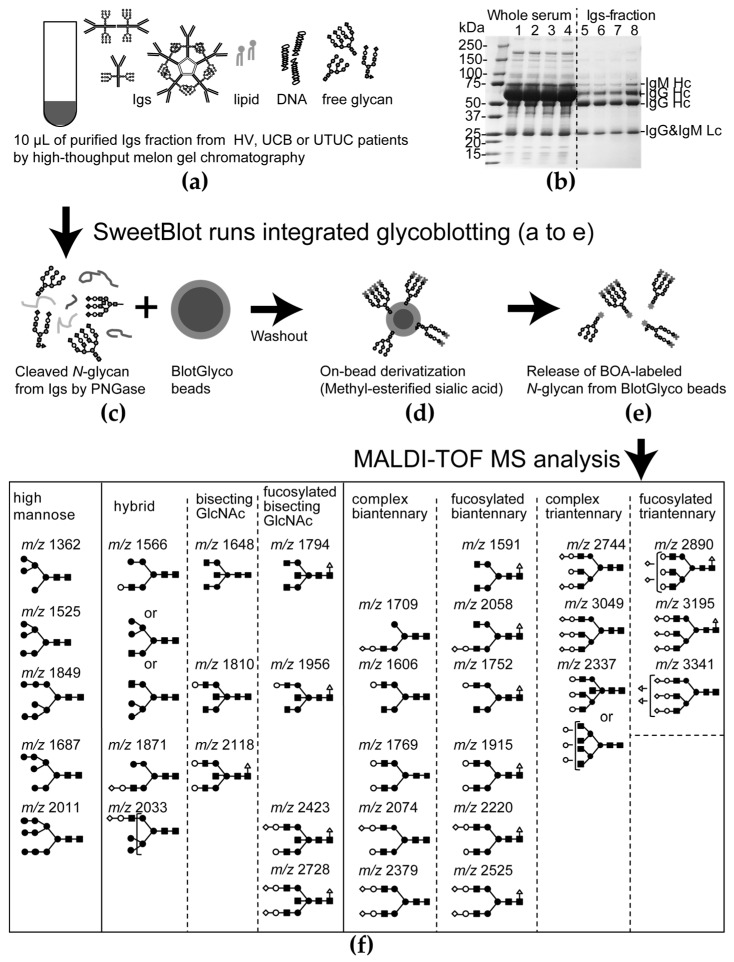
The general protocol for the integrated glycoblotting technique and workflow for glycoblotting-based high-throughput clinical glycan analysis. (**a**) Ten-microliter serum Ig fraction samples were applied to SweetBlot™ (System Instruments, Hachioji, Japan) for glycoblotting; (**b**) Coomassie brilliant blue (CBB)-stained band patterns on a sodium dodecyl sulfate-polyacrylamide gel for representative whole serum and purified Ig fraction samples; (**c**) After enzymatic *N*-glycan cleavage from Ig proteins, the total *N*-glycans released into the digestion mixture were directly mixed with BlotGlyco H beads (Sumitomo Bakelite, Co., Tokyo, Japan) to capture *N*-glycans; (**d**) After the beads were separated from other molecules by washing, sialic acid was methyl esterified; (**e**) These processed *N*-glycans were then labelled with benzyloxyamine (BOA) and released from BlotGlyco H beads; (**f**) Mass spectra of BOA-labelled *N*-glycans from Ig fractions were acquired using an Ultraflex III instrument (Bruker Daltonics, Germany). Total 32 type of *N*-glycans were identified in Igs fraction. Identified *N*-glycan structures are indicated by monosaccharide symbols: white circles, galactose (Gal); black circles, mannose (Man); black squares, *N*-acetylglucosamine (GlcNAc); black triangle, fucose (Fuc) and black diamonds, sialic acid.

SDS-PAGE analysis between whole serum (Figure A1b, lanes 1–4) and Igs fractions (Figure A1b, lanes 5–8) revealed that non-Igs proteins were effectively eliminated from whole serum by Melon Gel chromatography. *N*-glycomics of the Igs fraction (Figure A1a–e) identified 32 types of BOA-labelled *N*-glycans on Igs (Table A1, Figure A1f). Patient characteristics in the non-UC (HV and PC) and UC groups are shown in Table 1. There were no statistically significant differences in age, history of smoking, benign prostatic hyperplasia (BPH) and stone former between both non-UC group and UC group.

**Figure 1:**
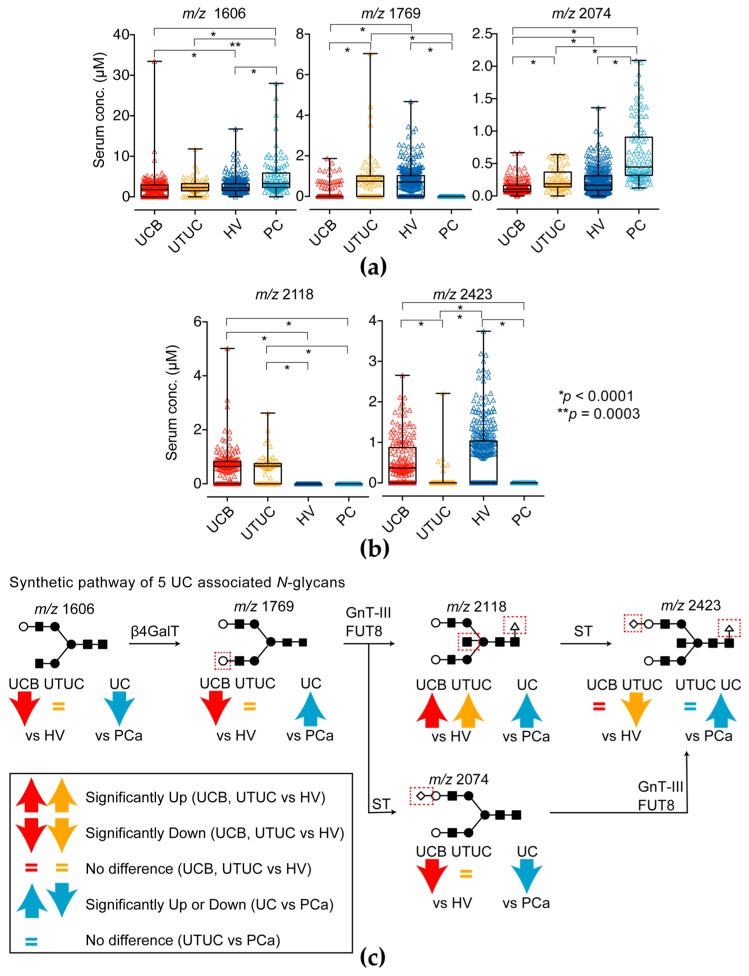
Five UC-associated *N*-glycan levels of immunoglobulins (Igs) fractions. (**a**) Complex biantennary-type *N*-glycan (*m*/*z* 1606, 1769 and 2074) levels and (**b**) fucosylated bisecting GlcNAc-type *N*-glycan (*m*/*z* 2118 and 2423) of the Ig fractions in the UC, HV and PC groups. The results shown are representative of three independent experiments. Intergroup differences were statistically compared using the Mann–Whitney *U*-test for non-normally distributed models; (**c**) Synthetic pathway of candidate *N*-glycans in the present study. Red up- and downward arrows indicates significantly changed in UC compare with HV group. Cyan up- and downward arrows indicates significantly changed in UC compare with PC group. The equals mark indicates that it did not significantly change. *N*-glycan structures are indicated by monosaccharide symbols: white circles, galactose (Gal); black circles, mannose (Man); black squares, *N*-acetylglucosamine (GlcNAc); black triangle, fucose (Fuc) and black diamonds, sialic acid. β4GalT: β1,4-galactosyltransferase, GnT-III: *N*-acetylglucosaminyltransferase-III, FUT8: fucosyltransferase 8, ST: sialyltransferase.

To detect UC, we performed multivariable discriminant analysis by inputting UCs event as an explanatory variable and the *N*-glycan level of Igs as objective variables and selected candidate *N*-glycans (complex biantennary type: *m*/*z* 1606, 1769, 2074; core fucosylated bisecting GlcNAc type: *m*/*z* 2118, 2423) that formed the most sensitive and specific combination for detection of UCs (Figure 1a–c and Table 2).

**Figure 2:**
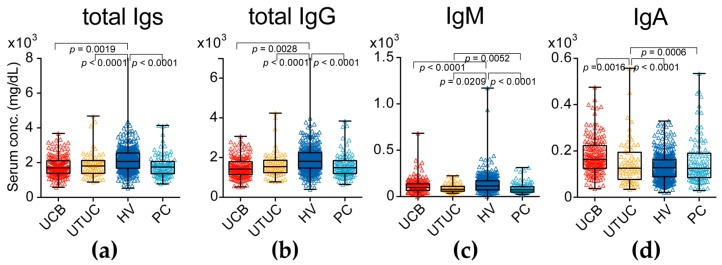
Serum immunoglobulin (Ig) levels in UC (UC of the bladder (UCB) and upper urinary tract UC (UTUC)) patients and healthy volunteers (HVs) and prostate cancer (PC). (**a**) Serum total Igs (sum of IgG1-4, IgM and IgA) levels in the UC, HV and PC groups; (**b**) Serum total IgG (IgG1-4) levels in the UC, HV and PC groups; (**c**) Serum IgM levels in the UC, HV and PC groups; (**d**) Serum IgA levels in the UC, HV and PC groups. The results shown are representative of three independent experiments. Intergroup differences were statistically compared using the Mann–Whitney *U*-test for non-normally distributed models.

The asialo biantennary type *N*-glycans (*m*/*z* 1606 and 2074) on Igs were significantly downregulated in the UC group compared with the levels in the HV and PC groups (*p* = 0.0001). Only the asialo biantennary type *N*-glycan (*m*/*z* 1769) on Igs was significantly downregulated in the PC group compared with the level in the UC and HV groups (*p* = 0.0001). The monosialyl bisecting GlcNAc with core fucosylated bisecting GlcNAc *N*-glycan (*m*/*z* 2423) was not significantly changed between UC and HV group, but significantly decreased in PC and UTUC group (*p* = 0.0001). Especially the asialo bisecting GlcNAc with core fucosylated *N*-glycan (*m*/*z* 2118) on Igs was significantly upregulated in UC group (*p* = 0.0001) but not detectable in HV and PC groups. However, total Igs level in UC patients was significantly lower than HV and PC patients (Figure 2a–d).

According to the synthetic pathway of *N*-glycans shown in Figure 1b and the above results, a downregulation of asialo biantennary type *N*-glycans and accumulation of asialo-bisecting GlcNAc with core fucosylated *N*-glycan on Igs may occur as a UC-associated aberrant *N*-glycosylation of Igs.

### 2.2. UC Diagnostic N-Glycan Score Based on Aberrant N-Glycosylation of Igs Was Far Superior to Classic Urine Cytology and Hematuria Status

To apply these candidate aberrant *N*-glycosylation profile on Igs for UC detection, we established the UC diagnostic *N*-glycan score (d*N*GScore) that was calculated according to the following formula:

d*N*GScore = (serum level of *m*/*z* 1606 × 0.1925) + (serum level of *m*/*z* 1769 × 0.4932) + (serum level of *m*/*z* 2074 × 0.4941) + (serum level of *m*/*z* 2118 × −3.2460) + (serum level of *m*/*z* 2423 × 0.6179) + (−0.4905) (1)

**Figure 3:**
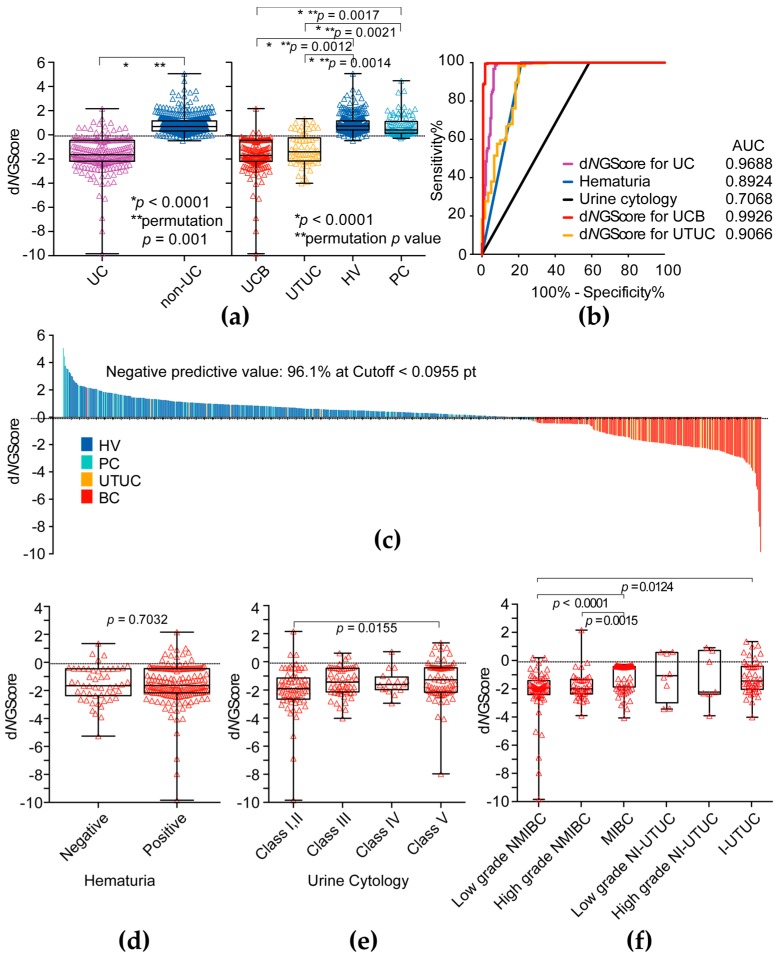
Clinical significance of diagnostic *N*-glycan score. (**a**) The diagnostic *N*-glycan score (d*N*GScore) level was significantly higher in the UC (UCB and UTUC) group than in the HV and PC group; (**b**) ROC curve analysis of d*N*GScore, hematuria and urine cytological results for detection of UC (UCB and UTUC); (**c**) Waterfall plot of d*N*GScore; (**d**) Association between d*N*GScore level and hematuria status; (**e**) Association between d*N*GScore level and urine cytology; (**f**) Association between d*N*GScore level and tumor invasiveness. NMIBC: non-muscle invasive bladder cancer, MIBC: muscle invasive bladder cancer, NI-UTUC: noninvasive UTUC, I-UTUC: invasive UTUC. Intergroup differences were statistically compared using the Mann–Whitney *U*-test for non-normally distributed models and performed permutation test.

The d*N*GScore was significantly lower in the UC (UCB and UTUC) patients than in the non-UC group including HVs and PC (Mann–Whitney *U*-test: *p* = 0.0001, permutation test: *p* = 0.001) (Figure 3a,c). The d*N*GScore of UCB was significantly lower than those of HV (Mann–Whitney *U*-test: *p* = 0.0001, permutation test: *p* = 0.012) and PC (Mann–Whitney *U*-test: *p* = 0.0001, permutation test: *p* = 0.0017) (Figure 3a). The d*N*GScore of UTUC was also significantly lower than those of HV (Mann–Whitney *U*-test: *p* = 0.0001, permutation test: *p* = 0.014) and PC (Mann–Whitney *U*-test: *p* = 0.0001, permutation test: *p* = 0.0021) (Figure 3a). The AUC of d*N*GScore for prediction of UCs (for UCs, 0.969, for UCB, 0.993, for UTUC, 0.907, respectively) was much higher than that of hematuria (AUC, 0.892) and urine cytology (AUC, 0.707) (Figure 3b and Table 3). At the cut-off d*N*GScore (−0.0955 points) for prediction of UCs, the negative predictive value (NPV) was 96.1%, which was much higher than the NPV of urine cytology (75.8%) and hematuria (89.5%). The d*N*GScore was not significantly associated with hematuria status and class of urine cytology (Figure 3d,e), but significantly associated with invasiveness of UCB (Figure 3f).

## 3. Discussion

Several studies have shown that differences in serum *N*-glycan profiles between diseased and benign states analyzing high-throughput, comprehensive and quantitative *N*-glycomics may be useful in the diagnosis or prognosis of diseases [11,12,13,14,19]. A few studies have investigated the use of serum *N*-glycans as diagnostic markers for UC, including UTUC [13,19]. Although these reports showed that the levels of highly branched sialylated *N*-glycans (*m*/*z* 2890, 3560, 3865) were increased in the sera of patients with bladder cancer [13,19], they did not identify carrier proteins of aberrant *N*-glycosylation. Furthermore, these highly branched sialylated *N*-glycans in serum were also significantly upregulated in plural cancer, such as prostate or kidney cancer [11,12]. To identify which carrier protein is aberrantly glycosylated, in the present study, we focused on *N*-glycomics of serum major *N*-glycosylated protein such as Igs. We showed that, in total, five types of *N*-glycans, including bisecting GlcNAc-, biantennary-type *N*-glycans with or without core fucose in serum Igs fractions, were associated with UC detection (Figure 1). According to the biosynthetic pathway of *N*-glycans (Figure 1c), bisecting GlcNAc *N*-glycans were synthesized from biantennary-type *N*-glycan by β1,4-*N*-acetylglucosaminyltransferase (GnT-III) and then modified terminal galactosylation and/or sialylation by galactosyltransferase and sialyltransferase. In this study, we found asialo biantennary- (*m*/*z* 1606 and 1769) and monosialyl biantennary-typed *N*-glycan (*m*/*z* 2074) and monosialyl bisecting GlcNAc-typed *N*-glycans (*m*/*z* 2423) were significantly decreased in UC patients and upregulated only asialo bisecting GlcNAc typed *N*-glycan (*m*/*z* 2118) on Igs in UC patients. Thus, we hypothesize asialo biantennary typed *N*-glycan on Igs was transformed to asialo bisecting type *N*-glycan on Igs by GnT-III activity and less sialyltransferase activity in UC patients and resulted in a decrease of biantennary and sialyl bisecting GlcNAc type *N*-glycan on Igs in UC patients. In addition, in the UTUC case, the level of monosialyl bisecting GlcNAc-typed *N*-glycans (*m*/*z* 2423) on Igs was significantly lower than that of UCB group. Thus, accumulation of asialo bisecting GlcNAc typed *N*-glycan (*m*/*z* 2118) was more of a UTUC-specific phenomenon than that of UCB. This suggests that aberrant *N*-glycosylation profile UTUC was little different from that of UCB. This difference may reflect the difference of tumor environment between bladder and urinary/renal pelvis, and/or may reflect different embryonic background of tumor origin. On the other hand, in PC case, upregulation of monosialyl biantennary-typed *N*-glycan (*m*/*z* 2074) and down-regulation of monosialyl bisecting GlcNAc-typed *N*-glycans (*m*/*z* 2423) is observed. This suggests that monosialyl biantennary-typed *N*-glycan (*m*/*z* 2074) on Igs is significantly accumulated in PC patients and might be the candidate aberrant glycosylation of prostate cancer detection. These aberrant *N*-glycosylation profiles of Igs were different from the whole serum aberrant *N*-glycosylation profiles obtained in previous studies [13,19]. Thus, asialo bisecting type *N*-glycosylated Igs can be applied as a promising UC-specific diagnostic biomarker. To the best of our knowledge, this is the first report to demonstrate the clinical significance of aberrant *N*-glycosylated Igs as diagnostic biomarkers of UCs. It is well known that glycosylation of Igs has a critical role in the development of diseases. Wuhrer et al. reported that asialo bisecting type *N*-glycosylated IgG induced anti-inflammatory response and agalactosyl bisecting type *N*-glycosylated IgG induced pro-inflammatory response in cerebrospinal fluid [20]. Overproduction of aberrantly glycosylated IgA1 has a key role in the development of IgA nephropathy [21]. A recent report suggested that antibody-mediated rejection after kidney transplantation is closely associated with the levels of immunomodulatory sialylated IgG antibodies [22]. Furthermore, Kazuno et al. reported that α2,6-sialylated IgG was significantly decreased in prostate cancer immunoreactions [24]. Rademacher et al. reported that agalactosyl bisecting GlcNAc type *N*-glycoslated IgG was increased in rheumatoid arthritis patient and related autoimmune disease [23]. From these observations, aberrant glycosylated Igs appear to change their glycans because of disease-associated immunoreactions. In a future study, we will examine *N*-glycosylation profile of Igs between benign disease and UC patients to address the UC associated aberrant glycosylation on Igs are part of inflammatory response.

In the present study, the d*N*GScore, combination of 5 *N*-glycans including biantennary and bisecting GlcNAc, clearly discriminate UC from healthy controls and prostate cancer patients with 92.8% sensitivity and 97.2% specificity. Our results also suggested that the level of d*N*GScore was not related to urine cytological classifications and hematuria (Figure 3d,e), which suggested a higher predictive value for urine cytology-negative (<Class IV) cases and hematuria negative cases. In addition, d*N*GScore can discriminate both low- and high-grade non-muscle invasive bladder cancer as well as muscle invasive bladder cancer. This suggests that the d*N*GScore may be useful for early diagnosis of UCB.

The limitations of the present study were its small sample size, retrospective nature, selection bias, lack of independent validation group and non-clinical setting. Therefore, the results obtained in this study have to be regarded as preliminary and need further validation study. Because urine cytological results are not reliable in patients with early stage UCs, including UTUC, a large-scale prospective validation study in a natural cohort of patients with hematuria is required. In addition, the usefulness of regular follow-up for detecting recurrence after surgery remains unclear; furthermore, a large-scale prospective follow-up study after surgery was also needed. In addition, this study did not include patients with benign diseases or infections such as calculi, UTI, cystitis, prostatitis. Despite these limitations, the strength of the present study was that it is the largest to assess the implications of aberrant *N*-glycosyleted Igs for UCs detection. Our findings may be useful for detection of UC patients and identification of patients at urine cytology negative UC. Furthermore, to apply the routine clinical practice, we now developed lectin-sandwich immunoassay system to detect aberrant asialo bisecting GlcNAc type *N*-glycosylated Igs as described previously [28].

## 4. Materials and Methods

### 4.1. Serum Samples

The present study was conducted in accordance with the ethical standards of the Declaration of Helsinki and approved by the institutional review board of Hirosaki University School of Medicine (“study about carbohydrate structure change in urological disease”; approval number: 2014-195, approval date: 22 December 2014). Written or verbal informed consent was obtained from all serum donors. Between 2009 and 2016, we randomly selected serum available 237 patients with UC and also 96 patients with prostate cancer as other cancer controls from our serum bank. Serum from any clinical treated patients were excluded. We also obtained from 339 healthy controls selected from community-dwelling volunteers in the health maintenance programme of Iwaki Health Promotion Project [19,29]. All serum samples were collected at the first visit and stored at ‒80 °C until use. All tumors were staged according to the 2017 tumor-node-metastasis classification, 8th edition [30]. Histological classification of UC was performed according to the World Health Organization 1973 and 2004 grading systems [31]. Urine cytology classification was performed according to guideline of The Paris System working group [32]. Patient demographics are shown in Table 1.

### 4.2. Purification and Quantification of the Igs Fraction from Serum

Each serum sample (100 μL) was applied to the center of the Zeba™ Spin desalting resin plate (Thermo Fisher Scientific, Waltham, MA, USA) equilibrated with phosphate-buffered saline and centrifuged at 1000× *g* for 2 min. The flow-through was collected as buffer-exchanged serum (100 μL). Purification of Igs fraction performed by using Melon™ Gel Spin Purification Kit (Thermo Fisher Scientific) according to the instructions. Buffer-exchanged serum (100 μL) was applied to the center of the Melon Gel resin equilibrated with purification buffer. After 5 min incubation, the Melon Gel resin was centrifuged at 1000× *g* for 2 min and the flow-through was collected as a purified Igs fraction. A 10-μL aliquot of the Igs fraction was subjected to *N*-glycomics. To confirm the purity of the Igs fraction, the Melon Gel flow-through Igs fraction was subjected to SDS-PAGE and stained with CBB. Igs (total IgG, IgM and IgA) levels of purified Igs fraction were measured by using a Bio-plex Pro Human Isotyping 6-plex kit (Bio-Rad Laboratories, Hercules, CA, USA) according to the instructions.

### 4.3. Serum N-Glycomics of Igs Performed by Using the Glycoblotting Method and Mass Spectorometric Analysis

*N*-glycomics was performed as described previously. A 10-μL aliquot of the purified Igs fractions were analyzed using the glycoblotting method [11,12,15,33] on a Sweetblot instrument (System Instruments, Hachioji, Tokyo, Japan). Then, the resulting benzyloxiamine (BOA)-labelled glycans were detected by matrix-assisted laser desorption time-of-flight (MALDI-TOF) mass spectrometry (Ultraflex 3 TOF/TOF mass spectrometer; Bruker Daltonics, Bremen, Germany) (Figure A1a–f). Composition and structures of the glycans were predicted using the GlycoMod Tool (available online: http://web.expasy.org/glycomod/) (Table A1). Quantitative reproducibility test of each *N*-glycans levels were then evaluated as described previously [19].

### 4.4. Statistical Analysis

Statistical analyses of clinical data were performed using SPSS v.22.0 (IBM Corporation, Armonk, NY, USA) and GraphPad Prism v.6.03 (GraphPad Software, San Diego, CA, USA). Categorical variables were reported as percentages and compared using the Fisher exact test. Age data were expressed as medians with 25th and 75th quartiles (Q1, Q3). Differences between the groups were statistically compared using the Student *t*-test for normally distributed data or the Mann–Whitney *U*-test for non-normally distributed data. Multivariable discriminant analysis for detection of UCs was performed by inputting UC event as an explanatory variable and *N*-glycan level as an objective variable. The diagnostic *N*-glycan score was calculated by multiplying candidate *N*-glycan levels by each discriminant function value. The diagnostic performance of *N*-glycan scores was evaluated using receiver operating characteristic (ROC) curve analysis developed using the library “rms’’ in R (available online: http://www.r-project.org/), and statistical differences between area under the curves (AUCs) were calculated using the same programme [34]. In order to assess the significant difference among two group of subjects we implemented a permutation tests and calculate permutation *p* value [35]. Differences with *p* < 0.05 were considered statistically significant.

## 5. Conclusions

In conclusion, aberrant *N*-glycosylation profiles of Igs determined by *N*-glycomics may be useful as diagnostic biomarkers for identifying UC patients. Future large-scale prospective validation studies are of vital importance.

## Figures and Tables

**Table 1:** Patients’ demographics in each cohort.

	Non-UC ^a^				UC ^b^	*p* Value a vs. b
	HV	*n*, (%)	PC	*n*, (%)	*n*, (%)	
Total patients (*n*)	339		96		237	
Sex (Male, %)	122 (36.0)		96 (100)		191 (80.6)	<0.001
Median age (IQR ^1^)	68.0 (63–73)		74.0 (68–78)		70.0 (62–75)	0.700
Former or current smoker	75 (22.1)		18 (18.8)		71 (29.9)	0.101
Stone former	4 (1.2)		0 (0)		0 (0)	0.184
BPH ^2^	7 (2.1)		0 (0)		0 (0)	0.145
HSPC ^3^	0 (0)		96 (100)		0 (0)	<0.001
hematuria+	0 (0)		0 (0)		186 (78.5)	<0.001
Urine Cytology Class						
I, II					81 (34.2)	
III					58 (24.5)	
IV					16 (6.7)	
V					82 (34.6)	
Tumor Location of UC						
Bladder					177 (67.6)	
Renal pelvis					27 (11.4)	
Ureter					28 (11.8)	
Multiple					4 (1.7)	
Tumor Grade of UC						
Low grade noninvasive					68 (28.7)	
High grade noninvasive					43 (18.1)	
Muscle invasive					109 (45.9)	
Lymph node stage N1			0 (0.0)		20 (8.4)	0.115
Metastatic disease			4 (4.2)		47 (19.8)	0.010

^1^ IQR, Interquartile range; ^2^ BPH, benign prostatic hyperplasia; ^3^ HSPC, hormone sensitive prostate cancer; HV: healthy volunteers; PC: prostate cancer. ^a^ non-urothelial carcinoma, non-UC; ^b^ urothelial carcinoma, UC.

**Table 2:** Multivariable discriminant analysis for prediction of UCs.

Variables	Wilks’ Lambda	*F* Value	ODF ^1^	TDF ^2^	*p* Value	Discriminant Function
*m*/*z* 1606	0.9742	17.72	1	670	<0.001	0.1925
*m*/*z* 1769	0.9707	20.24	1	670	<0.001	0.4932
*m*/*z* 2074	0.9377	44.54	1	670	<0.001	0.4941
*m*/*z* 2118	0.5894	466.73	1	670	<0.001	‒3.2460
*m*/*z* 2423	0.9984	1.04	1	670	<0.001	0.6179
				Constant term		‒0.4905

^1^ ODF, one degree of freedom; ^2^ TDF, two degrees of freedom.

**Table 3:** Comparing diagnostic performance of UCs on each test.

Variables	AUC	95% CI	Sensitivity (%)	Specificity (%)	PPV (%)	NPV (%)
Hematuria	0.892	0.861–0.924	78.5	100.0	100.0	89.5
Urine cytology	0.707	0.661–0.753	41.4	100.0	100.0	75.8
d*N*GScore ^1^ for UC	0.969	0.952–0.986	92.8	97.2	94.8	96.1
d*N*GScore for UCB	0.993	0.982–0.100	98.3	97.2	93.5	99.3
d*N*GScore for UTUC	0.907	0.854–0.959	77.1	97.2	79.7	96.8

^1^ d*N*GScore, diagnostic *N*-glycan score.

## Abbreviations

UC	Urothelial carcinoma
UCB	Urothelial carcinoma of the bladder
UTUC	Upper urinary tract urothelial carcinoma
HV	Healthy volunteers
PC	Prostate cancer
BPH	Beneign prostatic hyperplasia
HSPC	Hormone sensitive prostate cancer
NMIBC	Nonmuscle invasive bladder cancer
MIBC	Muscle invasive bladder cancer
NI-UTUC	Noninvasive UTUC
I-UTUC	Invasive UTUC
Igs	Immunogloblins
d*N*GScore	Diagnostic *N*-glycan Score
AUC	Area under the curve
ROC	Receiver operating characteristic curves
BTA	Bladder tumor antigen
NMP22	Nuclear matrix protein number 22
IQR	Interquartile range
NPV	Negative predictive value
PPV	Positive predictive value
UTI	Urinary tract infection
MALDI-TOF	Matrix-assisted laser desorption time-of-flight
Man	Mannose
Gal	Galactose
GlcNAc	*N*-acetylglucosamine
Fuc	Fucose
Sia	*N*-acetylneuraminic acid
CBB	Coomassie brilliant blue

## Appendix A

**Table A1:** Thirty-two types of *N*-glycans that showed good quantitative reproducibility in all Igs fraction samples and could be analyzed statistically.

#	*m*/*z*	Composition
1	1362.5	(Hex)2 + (Man)3(GlcNAc)2
2	1524.5	(Hex)3 + (Man)3(GlcNAc)2
3	1565.5	(Hex)5 + (HexNAc)3
4	1590.6	(HexNAc)2(dHex)1 + (Man)3(GlcNAc)2
5	1606.6	(Hex)1(HexNAc)2 + (Man)3(GlcNAc)2
6	1647.6	(HexNAc)3 + (Man)3(GlcNAc)2
7	1686.6	(Hex)4 + (Man)3(GlcNAc)2
8	1708.6	(Hex)1(HexNAc)1(NeuAc)1 + (Man)3(GlcNAc)2
9	1752.6	(Hex)1(HexNAc)2(dHex)1 + (Man)3(GlcNAc)2
10	1768.6	(Hex)2(HexNAc)2 + (Man)3(GlcNAc)2
11	1793.7	(HexNAc)3(dHex)1 + (Man)3(GlcNAc)2
12	1809.7	(Hex)1(HexNAc)3 + (Man)3(GlcNAc)2
13	1848.6	(Hex)5 + (Man)3(GlcNAc)2
14	1870.7	(Hex)2(HexNAc)1(NeuAc)1 + (Man)3(GlcNAc)2
15	1914.7	(Hex)2(HexNAc)2(dHex)1 + (Man)3(GlcNAc)2
16	1955.7	(Hex)1(HexNAc)3(dHex)1 + (Man)3(GlcNAc)2
17	2010.7	(Hex)6 + (Man)3(GlcNAc)2
18	2032.7	(Hex)3(HexNac)1(NeuAc)1 + (Man)3(GlcNAc)2
19	2057.8	(Hex)1(HexNAc)2(dHex)1(NeuAc)1+ (Man)3(GlcNAc)2
20	2073.8	(Hex)2(HexNAc)2(NeuAc)1+ (Man)3(GlcNAc)2
21	2117.8	(Hex)2(HexNAc)3(dHex)1 + (Man)3(GlcNAc)2
22	2219.8	(Hex)2(HexNAc)2(dHex)1(NeuAc)1 + (Man)3(GlcNAc)2
23	2336.9	(Hex)3(HexNAc)4 + (Man)3(GlcNAc)2
IS	2348.9 ^1^	Internal standard (BOA-labeled A2 amide)
24	2378.9	(Hex)2(HexNAc)2(NeuAc)2 + (Man)3(GlcNAc)2
25	2422.9	(Hex)2(HexNAc)3(dHex)1(NeuAc)1 + (Man)3(GlcNAc)2
26	2524.9	(Hex)2(HexNAc)2(dHex)1(NeuAc)2 + (Man)3(GlcNAc)2
27	2727.9	(Hex)2(HexNAc)3(dHex)1(NeuAc)2 + (Man)3(GlcNAc)2
28	2743.9	(Hex)3(HexNAc)3(NeuAc)2 + (Man)3(GlcNAc)2
29	2890.1	(Hex)3(HexNAc)3(dHex)1(NeuAc)2 + (Man)3(GlcNAc)2
30	3049.1	(Hex)3(HexNAc)3(NeuAc)3 + (Man)3(GlcNAc)2
31	3195.2	(Hex)3(HexNAc)3(dHex)1(NeuAc)3 + (Man)3(GlcNAc)2
32	3341.2	(Hex)3 (HexNAc)3 (Deoxyhexose)2 (NeuAc)3 + (Man)3(GlcNAc)2

^1^ *m*/*z* 2348.9 is the internal standard, disialo-galactosylated biantennary *N*-glycan, which contains amidated sialic acid residues (A2 amide glycans). Compositional annotations and putative structures are represented by the following abbreviations. Hex: hexose, HexNAc: *N*-acetylhexosamine, dHex: deoxyhexose.
